# College and Hospital Notes

**Published:** 1897-05

**Authors:** 


### College and Hospital Notes.

BELLEVUE Medical College and the New York University Medical College have consolidated their interests, the regents at Albany have sanctioned the fusion, and the united schools will hereafter be known as The New York University—Bellevue Hospital Medical College. The properties of both schools will be united and it is understood that the teachers in both will resign, leaving the council free to choose a new faculty from all available sources. It is hardly to be believed, however, that the council will find it necessary to go outside of the faculties of the two schools to supply a superb group of teachers for the reconstructed and consolidated school.

THE medical department of Niagara University will conclude its college year May 12, 1897. The alumni association will meet at the college building on that day, at 10.30 A. M., and hold both a morning and an afternoon session. An interesting program has been arranged. The commencement exercises will be held at the Star theater in the evening at 8 o'clock. Prof. John A. Miller, in behalf of the faculty, will deliver the valedictory, and Rev. F. S. Fitch, of Buffalo, will give the charge to the graduates. The alumni banquet will, as usual, follow the commencement exercises.

THE College of Physicians and Surgeons, at Chicago, has recently become the medical school of the University of Illinois. Thus, one by one, the creditable medical colleges are attaching themselves to universities. The personal or individual medical school will soon be a tradition.

